# MALT-1 shortens lifespan by inhibiting autophagy in the intestine of *C. elegans*

**DOI:** 10.1080/27694127.2023.2277584

**Published:** 2023-11-09

**Authors:** Julie Vérièpe-Salerno, Silvia Podavini, Marcus J.C. Long, Irina Kolotuev, Muriel Cuendet, Margot Thome

**Affiliations:** a Department of Immunobiology, Faculty of Biology and Medicine, University of Lausanne, Chemin des Boveresses 155, CH-1066 Epalinges, Switzerland; c Electron Microscopy Facility, University of Lausanne, Quartier Sorge – Biophore, CH-1015 Lausanne, Switzerland; d School of Pharmaceutical Sciences, University of Geneva, Rue Michel-Servet 1, CH-1211 Geneva, Switzerland; e Institute of Pharmaceutical Sciences of Western Switzerland, University of Geneva, Rue Michel-Servet 1, CH-1211 Geneva, Switzerland

**Keywords:** Aging, autolysosome, autophagosome, Beclin-1, chloroquine, electron microscopy, ATG-13, LGG-2, lifespan, starvation

## Abstract

The caspase-like protease MALT1 promotes immune responses and oncogenesis in mammals by activating the transcription factor NF-κB. MALT1 is remarkably conserved from mammals to simple metazoans devoid of NF-κB homologs, like the nematode *C. elegans*. To discover more ancient, NF-κB -independent MALT1 functions, we analysed the phenotype of *C. elegans* upon silencing of MALT-1 expression systemically or in a tissue-specific manner. MALT-1 silencing in the intestine caused a significant increase in life span, whereas intestinal overexpression of MALT-1 shortened life expectancy. Interestingly, MALT-1-deficient animals showed higher constitutive levels of autophagy in the intestine, which were particularly evident in aged or starved nematodes. Silencing of the autophagy regulators ATG-13, BEC-1 or LGG-2, but not the TOR homolog LET-363, reversed lifespan extension caused by MALT-1 deficiency. These findings suggest that MALT-1 limits the lifespan of *C. elegans* by acting as an inhibitor of an early step of autophagy in the intestine.

## Introduction

Mucosa-associated lymphoid tissue protein-1 (MALT-1, also known as paracaspase) is a caspase-like protease playing an essential role in mammalian immunity and oncogenesis. Humans and mice with mutations in the MALT1 gene show signs of severe immunodeficiency, often combined with autoimmunity, whereas hyperactivity of MALT1 is associated with cancers of both hematopoietic and non-hematopoietic origin [1–3]. The molecular function of MALT1 has been intensively studied. It has become clear that MALT1 exerts a scaffold function by binding to other signaling proteins, but also an enzymatic function, by cleavage of a variety of cellular substrates [1,2,4]. MALT1 is typically activated by triggering of cell surface receptors on immune cells, such as the B- and T-cell antigen receptor or the innate immune receptor Dectin-1 [5]. Other receptors that signal via MALT1 in non-immune cells are G-protein coupled receptors (GPCRs) and the epidermal growth factor receptor (EGFR) [5]. A common feature of these receptors is their capacity to induce the formation of oligomeric signaling complexes comprising a caspase-recruitment domain (CARD)-containing scaffold protein, the adaptor protein B-cell lymphoma-10 (BCL10), and MALT1. The assembly of these so-called CBM complexes enables MALT1-dependent downstream signaling events, including the recruitment of ubiquitin ligases like TRAF6 [6] and LUBAC [7], which activate the transcription factors nuclear factor NF-kappa B (NF-κB) and activator protein-1 (AP-1) to drive inflammatory and proliferative cell responses. Oligomerized MALT1 also becomes proteolytically active and cleaves a series of cellular substrates, boosting activation of NF-κB and AP-1 and prolonging the stability of a subset of otherwise short-lived gene transcripts [8,9]. Collectively, these studies have led to the concept that MALT1 promotes inflammatory immune responses and tumorigenesis in mammals mainly through effects at the mRNA level.

A few studies have suggested that MALT1 exerts additional, NF-κB and transcription-independent functions that remain less well understood. MALT1 regulates for example T-cell adhesiveness, by cleavage of its binding partner BCL10 [10], and promotes endothelial permeability by cleavage of the deubiquitinating enzyme CYLD, which results in microtubule destabilization [11]. Further indirect support for the existence of NF-κB-independent functions of MALT1 comes from the observation that the MALT1 gene is highly conserved from mammals to simple metazoans devoid of NF-κB homologs [12,13]. Indeed, the *C. elegans malt-1* gene conserves most of the functional domains found in its human homolog, including the protease domain. However, *C. elegans* lacks homologs of BCL10 and the CARD-containing upstream regulators, considered indispensable for MALT1 activation in mammalian cells [13].

MALT-1 mutation in *C. elegans* allele tm289 is lethal or sterile, but this strain contains a 973 bp deletion in the MALT-1-encoding gene *malt-1* (F22D3.6), extending into the neighboring gene *ceh-38* (F22D3.1). CEH-38 is a homeobox protein that regulates transcription by RNA polymerase II; the tm289 allele may thus not reflect the phenotype of a *bona fide malt-1* knock-out (Wormbase phenotype database). Indeed, RNAi-based silencing of *malt-1* systemically or preferentially in neurons does not impact viability but shows a significant reduction in lifespan upon *malt-1* silencing in neurons [13]. In addition, MALT-1 is involved in neuronal IL-17 signaling, which regulates escape behavior of the nematode to surface oxygen exposure as well as aspects of associative learning and immunity [14].

The lifespan of *C. elegans* is affected by a variety of elements, including environmental factors such as temperature, food availability and oxygen levels, but also genetic alterations affecting vital processes such as reproduction, uptake and sensing of nutrients, and protein/organelle turnover by macroautophagy (referred to as autophagy hereafter) [15,16]. Autophagy is a cellular process that is vital for the elimination of intracellular molecules and cellular substructures by targeting these for lysosomal degradation [17]. It plays a central role in several longevity pathways and contributes to prolonging the lifespan of *C. elegans*, but also of other organisms such as flies and mice, by promoting degradation and recycling of cellular components, which renders the animals more resistant to starvation [18,19]. Dietary restriction promotes the expression of autophagy genes through various nutrient sensing pathways that induce autophagy regulators via transcriptional and epigenetic changes [20]. The lifespan of *C. elegans* is shortened by mutation or RNAi-mediated silencing of essential autophagy-related (ATG) genes, such as *unc-51, bec-1, lgg-1*, or *atg-9* [19,21,22]. In contrast, treatment with autophagy activators such as the TORC1 inhibitor rapamycin or the polyamine spermidine, or upregulation of autophagy genes by overexpression of the transcription factor HLH-30 (a TFEB ortholog), increases longevity [23–26].

Here, we used mutational and gene silencing approaches to inhibit the activity or expression of MALT-1 in *C. elegans* and observed a role of intestinal MALT-1 in limiting the longevity of *C. elegans*. MALT-1 deficiency significantly prolonged *C. elegans* lifespan, particularly under conditions of starvation. The lifespan extension induced by MALT-1 deficiency correlated with an increase in autophagy and was reverted by silencing of autophagy regulators ATG-13, BEC-1, or LGG-2 (ATG8), or by chloroquine treatment. Our findings suggest that MALT-1 limits the lifespan of *C. elegans* by inhibiting an early step of autophagy vital for the survival of aging or starved animals.

## Results

### MALT-1 is expressed in the nervous system and digestive tract of C. elegans

**Figure 1:**
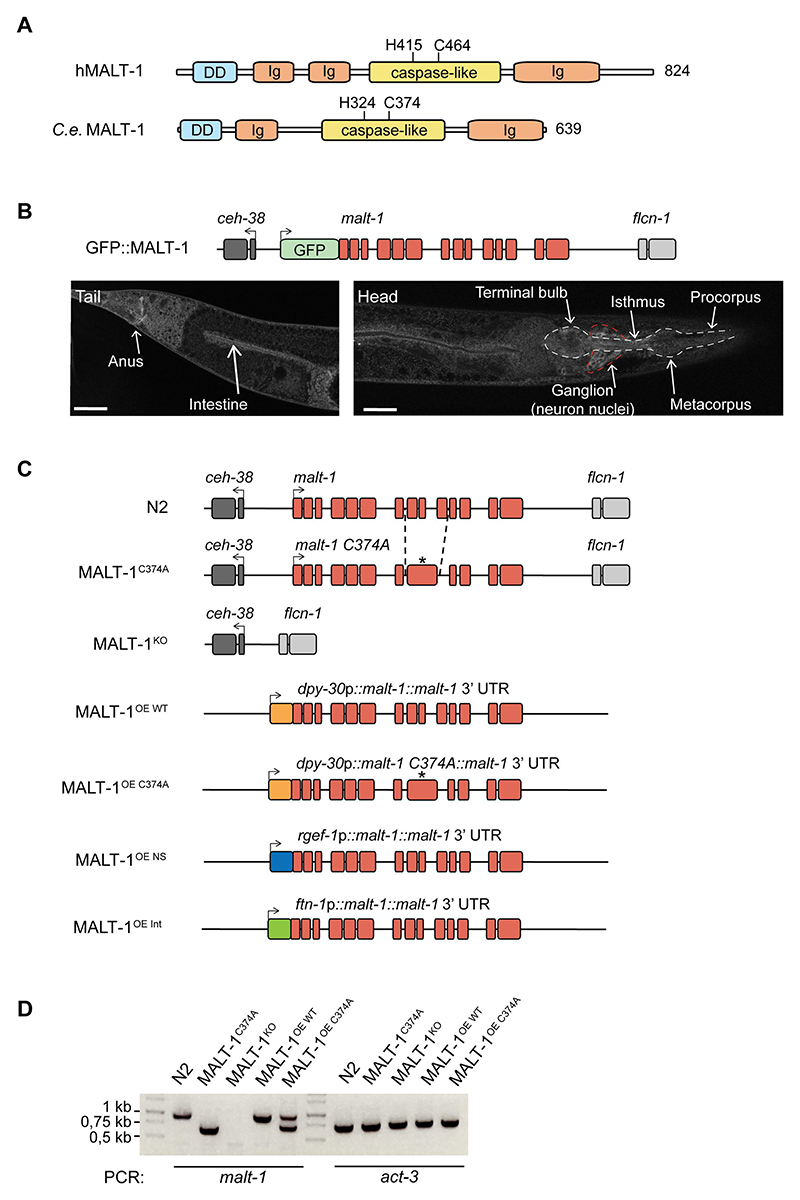
Generation and basal characterization of MALT-1 mutants in *C. elegans*. (A) Overview of the protein structure of human and *C. elegans* MALT-1, comprising a death domain (DD), immunoglobulin (Ig) domains and a caspase-like protease domain (caspase-like). The positions of the active site histidine and cysteine residues are indicated. (B) Illustration and confocal imaging of the GFP::MALT-1 construct used to visualize MALT-1 expression. The construct was generated by a 5’ in-frame insertion of GFP into exon 1, replacing the natural start codon of *malt-1* by the last codon of GFP. Confocal imaging shows GFP::MALT-1 expression in a young animal. MALT-1 is mostly expressed in the annotated structures. The white dotted line represents the pharynx and the red dotted line the nerve ring. Scale bar: 25 μm. (C) Overview of the *malt-1* gene structure in the wild-type (N2) and mutated strains generated for this study. *malt-1* exons are shown in red. The position (*) of the inactivating gene mutation and the exon structure of the *malt-1* gene, where a cysteine at the position 374 is mutated to an alanine (C374A), is illustrated. The strains overexpressing MALT-1 (MALT-1^OE^, MALT-1^OE NS^, MALT-1^OE Int^) were generated by insertion of the *malt-1* gene with its 3’UTR next to the gene promoters dpy-30, rgef-1 and ftn-1, driving strong expression in a ubiquitous manner (OE), in the nervous system (OE NS) and the intestine (OE Int), respectively. Arrows indicate the reading sense of the *malt-1* and flanking genes. (D) PCR analysis of the indicated *C. elegans* strains, monitoring presence or absence of wild-type or mutated *malt-1* and *act-3* (a housekeeping gene, used as a control for equal DNA content) for each indicated strain.

The human and *C. elegans* MALT-1 proteins have similar overall structures, comprising an N-terminal death domain and a central caspase-like protease domain, followed by an adjacent immunoglobulin-like (Ig) domain (Figure 1A). The human MALT-1 protease active site comprises a histidine at position 415 (H415) and a cysteine at position 464 (C464), which are essential for catalytic activity. This dyad is conserved in the *C. elegans* protein. In contrast, the presence of two Ig domains preceding the protease domain, which are responsible for BCL10 binding in human MALT-1, is not conserved in *C. elegans*. This likely reflects the fact that *C. elegans* has no BCL10 homolog and therefore does not rely on BCL10-binding for MALT-1 activation. The absence of a C-terminal extension, which contains TRAF6 binding sites and an autoprocessing site in human MALT1, additionally suggests differences in the overall regulation and function of *C. elegans* MALT-1. To gain insight into the function and tissue expression of MALT-1, we generated a strain expressing a green fluorescent protein (GFP)-MALT-1 fusion protein from the endogenous *malt-1* promoter (GFP::MALT-1) (Figure 1B). The parental *C. elegans* strain N2 shows strong autofluorescence, particularly in the intestine, which may mask the detection of the GFP::MALT-1 fusion (Figure S1A). We therefore crossed the GFP::MALT-1 strain with the *glo-1(zu391)* mutant, which has a globally weaker autofluorescence intensity in the intestine [27] (Figure S1B). Using this strain, and consistent with previously reported data [14], we detected MALT-1 expression in the digestive system – the pharynx, intestine, and anal region – and in the nerve ring (Figure 1B, S1C and S1D). We also observed that GFP fluorescence intensified at day 7 of adulthood compared to day 2, suggesting that MALT-1 expression increases with aging (Figure S1B).

### MALT-1 is not required for the viability and fertility of C. elegans

To further assess the role of the activity and expression of MALT-1 in *C. elegans*, we generated *C. elegans* strains with a protease inactivating C374A point mutation (MALT-1^C374A^) or a full deletion of the *malt-1* gene (MALT-1^KO^) in the N2 wild-type background (Figure 1C). Furthermore, we generated strains that ubiquitously overexpress (OE) wild-type or protease inactive MALT-1, using the Mos1 transposon-based technology to insert the *malt-1* gene - or its mutated C374A version - as a single copy under the control of the *dpy-30* promoter (Figure 1C). In addition, we generated strains that overexpress MALT-1 in the nervous system (MALT-1^OE NS^), under the control of the *rgef-1* promoter, or in the intestine (MALT-1^OE Int^), under the control of the *ftn-1* promoter (Figure 1C). All genotypes were verified by PCR (Figure 1D) and sequencing (not depicted), and absence of malt-1 mRNA in the MALT-1^KO^ strain was controlled by RT-PCR (Figure S1C). Because the *malt-1(tm289)* strain has been reported to be lethal or sterile, we also assessed the fertility of these nematodes: overall, we noticed no significant changes in the number of progeny compared to the N2 strain (Figure S1D). Thus, alterations in the expression or catalytic activity of MALT-1 do not affect the viability and fertility of *C. elegans*.

### Intestinal MALT-1 expression limits the lifespan of C. elegans

**Figure 2:**
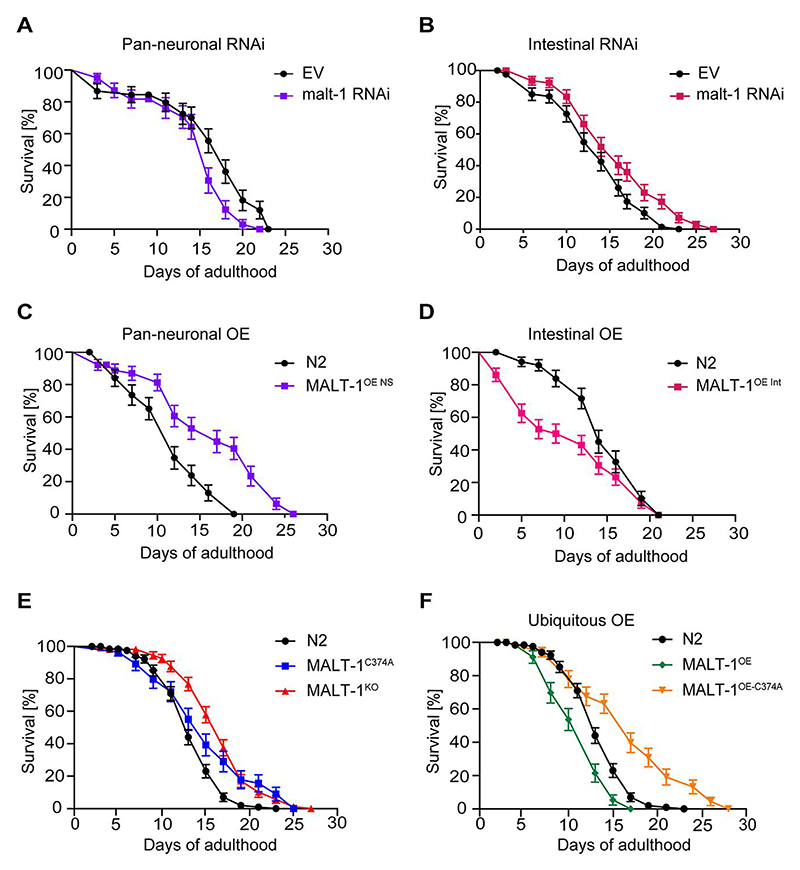
Intestinal MALT-1 expression shortens the lifespan of *C. elegans*. (A, B) Lifespan of pan-neuronal (A) and intestinal (B) RNAi-sensitive strains on empty vector (EV) or malt-1 RNAi-containing bacteria, at 20°C. The pan-neuronal RNAi and the intestinal RNAi curves were significantly different from EV-fed animals, p-values <0.05 and <0.01 respectively. (C, D) Lifespan of strains overexpressing wild-type MALT-1 in the nervous system (NS) under the regf-1 promoter (C) and in the intestine (Int) under the ftn-1 promoter (D). MALT-1^OE NS^ and MALT-1^OE Int^ curves were significantly different from N2, p-values <0.0001 and <0.01 respectively. Lifespan was measured for the indicated strains at 20°C on OP50. (E-F) Comparison of the lifespan of N2 with the catalytically inactive strain MALT-1^C374A^ and the *malt-1* deleted strain MALT-1^KO^ (E) or with the ubiquitously *malt-1* overexpressing strain MALT-1^OE^ and its inactive version MALT-1^OE-C374A^ (F), on NGM plates streaked with OP50. P-values were <0.01 for the MALT-1^C374A^ curve and <0.0001 for the others. Results of one representative experiment performed in triplicate are depicted, with an average of 75 nematodes per condition. All graphs are representative of three independent experiments. The log-rank Mantel-Cox test was used to determine p-values.

Previous studies have reported conflicting roles for neuronal versus non-neuronal MALT-1 in *C. elegans* longevity. One study reported that RNAi-mediated *malt-1* silencing in neurons shortened the lifespan of *C. elegans*, while silencing of *malt-1* in non-neuronal tissue showed no effect on lifespan [13]. Another study observed an increase in the lifespan of MALT-1-deficient *C. elegans* animals, which was reverted by pan-neuronal MALT-1 expression [14]. This suggests that MALT-1 can affect *C. elegans* lifespan in a manner that is tissue- and context-specific. Based on the expression of MALT-1 in both, neuronal and intestinal tissues (Figure 1B), we investigated tissue-specific roles of MALT-1 in lifespan regulation, by assessing the effects of *malt-1* silencing in neurons *versus* the intestine. For this, we fed animals with HT115 bacteria containing an empty vector (EV) or a *malt-1*-specific RNAi construct, using a *C. elegans* strain in which only pan-neuronal tissue or only intestinal tissue was sensitive to RNAi (TU3401 or MG171, respectively). In both instances, efficient but not complete reduction of *malt-1* mRNA was observed by RT-PCR (Figure S2A). Silencing of *malt-1* in neuronal cells decreased lifespan compared to control, consistent with previous findings [13] (Figure 2A). Conversely, RNAi-mediated *malt-1* silencing in the intestine increased longevity compared to control (Figure 2B). As a corollary, neuronal MALT-1 overexpression increased lifespan, whereas intestinal MALT-1 overexpression shortened lifespan (Figure 2, C and D; Tables S1 and S2). We also assessed the lifespans of MALT-1^KO^ and MALT-1^C374A^ strains (Figure 2E and Table S3), and of strains ubiquitously overexpressing either wild-type MALT-1 or its catalytically inactive C374A mutant (Figure 2F and Table S3). Under conditions of standard feeding with live *E. coli* OP50 bacteria, the MALT-1^KO^ and protease inactive MALT-1^C374A^ strains both showed an extended lifespan compared to the N2 strain (Figure 2E and Table S3). Ubiquitous overexpression of wild-type MALT-1, on the other hand, shortened lifespan, whereas ubiquitous overexpression of protease inactive MALT-1 extended lifespan (Figure 2F and Table S3). Thus, catalytically active MALT-1 limits *C. elegans* longevity, mainly by effects on the intestine.

Given the role of human MALT-1 in the immune response [1], we considered the possibility that the observed lifespan differences (Figure 2 and Tables S1-3) are due to OP50 pathogenicity [28]. We thus tested the longevity of different MALT-1 mutant strains upon feeding with heat-killed bacteria that show reduced pathogenicity [29]. Feeding the nematodes heat-killed bacteria increased the longevity of N2 animals, but deficiency or mutational inactivation of MALT-1 nonetheless increased lifespan compared to the N2 strain (Figure S2B). Collectively, these findings suggest that systemic or intestinal lack of MALT-1 extends *C. elegans* lifespan, and that the protease activity is essential for MALT-1-dependent lifespan limitation.

### MALT-1 deficiency renders C. elegans resistant to starvation

**Figure 3:**
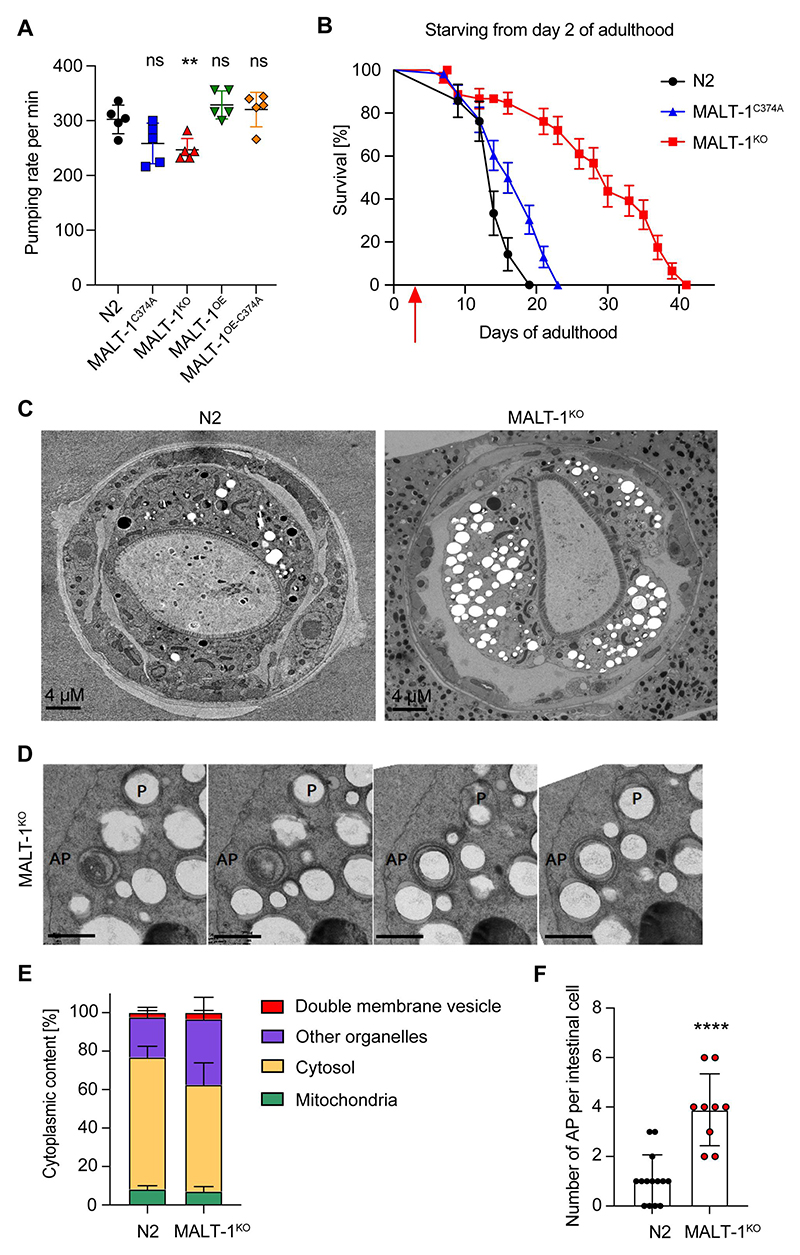
MALT-1^KO^ animals are more resistant to starvation and have higher numbers of autophagosomes than N2. (A) Pharyngeal pumping rate of the indicated strains at 18°C. ** p <0.01; ns, not significant (unpaired t-test). (B) Lifespan test for the indicated starved strains at 20°C. Animals were synchronized at L4 on NGM plates streaked with OP50 until day 2 of adulthood and then transferred (red arrow) onto plates without food and supplemented with ampicillin to avoid contamination. (C) Electron microscopy of synchronized N2 and MALT-1^KO^ animals following starvation for seven days after reaching adulthood. (D) Z-stack of a double membrane vesicle identified as autophagosome (AP). A phagophore (P) surrounding a vesicle is also present. Scale bars: 500 nm. (E) Stereological quantifications of pictures from three N2 and four MALT-1^KO^ animals starved for 7 days. Percentages of double membrane vesicles, mitochondria, cytosol and other organelles within the imaged intestinal cells (excluding intestinal lumen and nuclei). Significant differences were observed for double membrane vesicles (*, p<0.05) and other organelles (**, p<0.1) in N2 and MALT-1^KO^, using a Mann-Whitney test. (F) Quantification of the number of autophagosomes per intestinal cell. Autophagosomes were counted from three N2 and four MALT-1^KO^ animals, from which multiple images were taken, n=14 for N2 vs n=9 for MALT-1^KO^. Unpaired t-test was applied (****, P<0.0001). Pharyngeal pumping rate in (A) is representative of three independent experiments. One example graph from four separate tests is shown for the lifespan after food deprivation in (B). For electron microscopy (C), images were taken from multiple animals at day 7 of adulthood.

Previous studies have shown that dietary restriction extends the lifespan of *C. elegans* [30,31]. Therefore, we wondered whether the extended lifespan of *malt-1*-deficient animals resulted from a defect in food uptake. The pharynx is part of the alimentary system responsible for food pumping and grinding activities. When analyzed by transmission electron microscopy (TEM), MALT-1-deficient animals showed no obvious changes in pharyngeal structure (Figure S3A). Next, we measured the pharyngeal pumping rates of our strains and observed that MALT-1^KO^ animals showed an approximately 15% reduction in the pumping rate compared to N2. However, no significant changes in pumping rates were apparent in strains expressing catalytically inactive MALT-1 or overexpressing wild-type or inactive MALT-1 (Figure 3A). Thus, the reduction in pharyngeal pumping in the MALT-1-deficient strain is unlikely to account for its increased lifespan.

To assess whether MALT-1 plays a potential role in the resistance of *C. elegans* to food restriction, we grew animals on plates with low peptone content, which limits bacterial growth and thus nutrient availability [32,33]. This extended the lifespan of N2 animals, which was further enhanced by the absence or catalytic inactivation of MALT-1 (Figure S3B). Next, we performed a complete starvation of synchronized animals starting on day 2 of adulthood. Unlike dietary restriction, which prolongs the lifespan, complete starvation did not prolong lifespan in N2. However, MALT-1-deficiency dramatically increased the lifespan of the starved animals (Figure 3B). Conversely, nematodes expressing a protease inactive MALT-1 showed only a mild increase in lifespan. These findings suggest that MALT-1, predominantly via its scaffold function, restricts *C. elegans’* capacity to survive during starvation.

### MALT-1-deficient animals have more vesicular structures and autophagosomes

We proceeded to compare subcellular structures present in intestinal cells of fed and starved N2 and MALT-1^KO^ animals by TEM. Analyses of fed L4 animals did not reveal any striking differences between wild-type and MALT-1^KO^, except for the presence of a small number of double-membrane vesicles in the MALT-1^KO^ but not the wild-type strain (Figure S3C). Given that starving augmented the longevity promotion of MALT-1^KO^ (Figure 3B), we starved adult N2 and MALT-1^KO^ animals for 7 days and analyzed animals by TEM. The volume of the pseudocoelom appeared to be more prominent in MALT-1^KO^ compared to N2 (Figure 3C). Moreover, we observed a higher number of vesicular structures, often surrounded by swollen rough endoplasmic reticulum (RER) in MALT-1^KO^ (Figure S3D). To quantify these differences, we decided to stereologically quantify cellular components, and especially double membrane vesicles in EM pictures obtained from the anterior part of the intestine of three and four individual N2 and MALT-1^KO^ animals, respectively (Figure 3D, 3E, and S4). The analysis of consecutive TEM images allowed us to identify some double membrane vesicles as phagophores or autophagosomes (AP) (Figure 3D). Mitochondria were used as control organelles in our quantification, since in both strains they were represented in similar proportions (Figure 3E). We also quantified the relative volumes of cytosol and of a group of membrane-surrounded structures that we defined as “other organelles”, which comprise Golgi and ER structures, single membrane vesicles, lipid vesicles, and other unidentified structures (Figure 3E). This quantification revealed a small but significant increase in the percentage of double membrane vesicles and more generally an increase of other membrane-surrounded organelles in MALT-1^KO^ versus N2 (Figure 3E and S3C). We then analyzed double membrane vesicles in more detail. When comparing intestinal cells (not stereologically) in N2 *vs* MALT-1^KO^ we observed that the number of APs was drastically increased in MALT-1-deficient animals (Figure 3F and S4). These results suggest that, compared to wild-type controls, MALT-1-deficient animals have increased autophagic activity after 7 days of starvation, which might contribute to the remarkable resistance of MALT-1-deficient nematodes to food deprivation.

### MALT-1-deficient animals show increased autophagy reporter activity during aging and starvation

**Figure 4:**
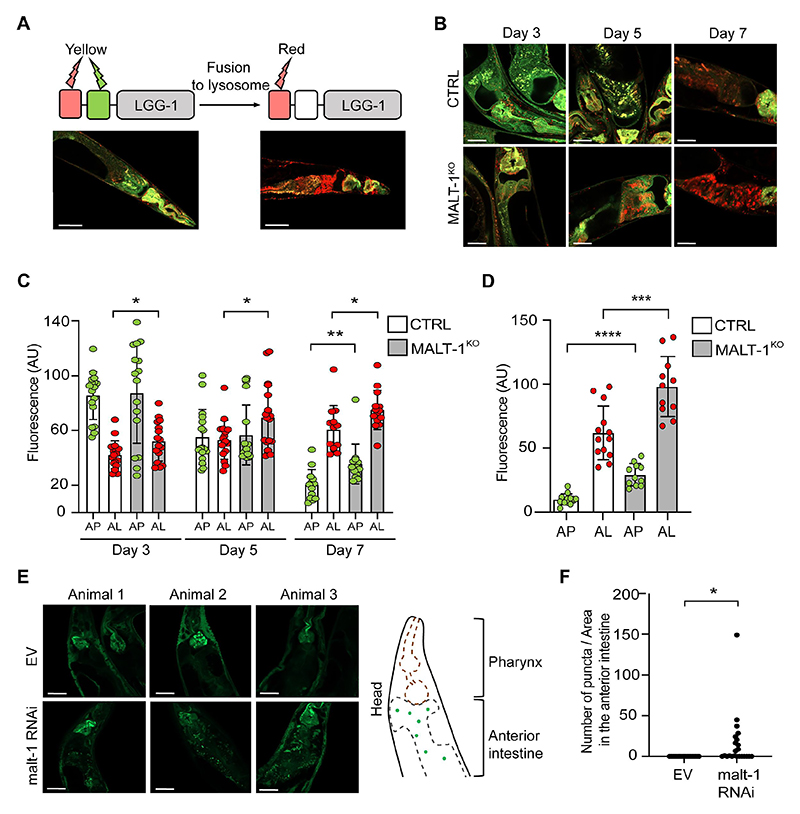
MALT-1 deficiency leads to increased autophagy during aging or under conditions of starvation. (A) Scheme representing the function of the GFP-mCherry-LGG-1 reporter strain used for monitoring autophagy. The fluorescence of GFP is quenched when its environment becomes acidic, such as upon fusion of autophagosomes (yellow) with lysosomes during formation of an autolysosome (red). Representative pictures of the strain under condition of aging at day 5 (left) and day 7 (right) are shown. Scale bars: 50 μm. (B-D) Fluorescence analysis of the anterior intestine of N2 and MALT-1^KO^ strains, crossed to the GFP-mCherry-LGG-1 reporter strain, at days 3, 5 and 7 of adulthood. Representative pictures of *C. elegans* (B) and quantification of fluorescence intensities (C) are shown. AP, autophagosomes (yellow fluorescence); AL, autolysososme (red fluorescence); AU, arbitrary units. Unpaired t-test was used for statistical analysis (*, p<0.05; **, p<0.01). Scale bars: 25 μm. (D) Quantification of fluorescence intensities in control (CTRL) *vs* MALT-1^KO^ animals starved for 96 h. Relative fluorescence intensity of autophagosomes (AP) and autolysosomes (AL) is indicated by green and red dots, respectively. AU, arbitrary units. Unpaired t-test was used for statistical analysis (***, p=0.0006; ****, p<0.0001). The complete graph including fed conditions is shown in Figure S4. (E, F) Three representative confocal pictures of living GFP::LGG-1 animals, fed with EV or *malt-1*-specific RNAi and then starved for 7 days. Scale bars: 25 μm. GFP aggregates in the anterior intestine (E, right panel) were counted in ten animals (F). The puncta number was standardized based on the size of the anterior intestine imaged and expressed as a density (F). A t-test was used, and the p-value is indicated. The experiment was done three independent times.

To further assess how MALT-1 deficiency affects autophagy, we used a previously described *C. elegans* strain, MAH215, expressing an *lgg-1p:: mCherry::gfp::lgg-1* reporter construct that serves as a reporter for autophagy [34]. The gene *lgg-1* (also known as *atg8.1*) encodes the autophagy protein LGG-1, a *C. elegans* homolog of mammalian GABA type A receptor-associated protein (GABARAP). During the initiation of autophagy, LGG-1 is lipidated, recruited to the growing phagophore membrane and distributed on the surface of the outer and inner AP double membrane. At this stage, mCherry::GFP::LGG1 gives a yellow fluorescent signal due to mCherry and GFP both being active. Fusion of APs with lysosomes yields autolysosomes (AL), lowering the pH of the vesicle content and quenching GFP fluorescence. At the AL stage, the fluorescence emission of the reporter is thus dominated by the red fluorescence of mCherry. Autophagic activity in worms decreases with age in the intestine, resulting in an enrichment of AL over AP [34]. The latter is likely due to an incapacity to complete the autophagic process because of reduced lysosomal acidification in old animals, which delays cargo degradation [35]. Animals containing mainly AP (such as 3 days old animals) or AL (such as 7 days old animals), can therefore be distinguished by fluores-cence microscopy (Figure 4A) and the relative fluorescence can be quantified in the intestine. To explore whether MALT-1 deficiency affects age-dependent changes in autophagy, we crossed the MAH215 strain containing the fluorescent reporter with the MALT-1^KO^ strain and monitored the presence of AP and AL by fluorescence microscopy at days 3, 5 and 7 of adulthood in the anterior intestine (Figure 4, B and C). To be able to compare nematodes of different ages, we took confocal pictures using the same settings. However, upon aging, fluorescence intensity increased to a level that made it impossible to distinguish individual puncta. To avoid underestimation of the number of puncta, we therefore decided to count global fluorescence, assuming that autofluorescence would be similar in the control and in the MALT-1^KO^. During the aging process from day 3 to day 7, we observed a continuous increase in the relative detection of AL and a corresponding decrease in AP in both, the *malt-1*-deficient and wild-type strain. However, at every time point, higher levels of AL were present in the MALT-1^KO^ strain versus the control; this difference remained stable over time. At day 7 of adulthood, we additionally observed a significantly increased yellow fluorescence intensity, suggesting that at this age MALT-1^KO^ also had more AP than the control strain. Our results are consistent with the observations reported by Chang *et al*. for wild-type *C. elegans* [34], and indeed suggest that *malt-1* null animals have enhanced autophagy during ageing, which could contribute to the increased lifespan of the MALT-1^KO^ strain.

Since the MALT-1^KO^ strain was highly resistant to starvation (Figure 3B), we wondered whether this phenotype correlates with altered starvation-induced autophagy levels. We used the same MAH215 reporter strain to monitor the presence of AP and AL after 96 h starvation, in animals fed with bacteria expressing empty vector or MALT-1-specific RNAi. In the fed condition, we observed no statistical differences in the mean fluorescence intensities between the control and MALT-1-silenced strain but noticed a tendency toward more AP and AL in MALT-1^KO^ compared to wild-type nematodes (Figure S5A). However, differences between the MALT-1^KO^ and control strains became statistically different in the starved condition, where AP and AL pools in MALT-1^KO^ strain were larger than in the N2 control (Figure 4D). We also assessed autophagy in the MAH215 reporter strain, by monitoring expression of the mCherry::GFP::LGG-1 fusion protein by western blotting for GFP. Upon 5 days of starvation, we observed a mild but reproducible reduction of the total levels of the mCherry::GFP::LGG-1 fusion protein by western blot, indicating starving-induced autophagy, as previously reported [36] (Figure S5B). This reduction was much more pronounced in the corresponding MALT-1^KO^ strain (MTM38), suggesting a further increase in the total level of autophagy and subsequent lysosomal turnover of the LGG-1 fusion protein in the absence of MALT-1. To verify our results in an alternative system, we made use of a strain expressing a GFP::LGG-1 construct (DA2123), which allowed visualization and quantification of LGG-1 puncta, instead of global fluorescence, generated after autophagy engagement. In these nematodes, we silenced *malt-1* gene expression by RNAi for 24 h and then starved the animals for 7 days to induce autophagy. Efficiency of *malt-1* silencing was verified after 5 or 7 days of starvation by RT-PCR (Figure S5C). Counting GFP::LGG-1 puncta in the anterior intestine by confocal microscopy revealed the presence of a higher density of GFP::LGG-1 aggregates upon *malt-1* knock-down compared to the EV condition (Figure 4, E and F). Collectively, these findings suggest that MALT-1 acts as an inhibitor of autophagy in the intestine, thereby preventing autophagy-dependent adaptations to aging and starving.

### Inhibition of autophagy abolishes lifespan extension by MALT-1 deficiency

**Figure 5:**
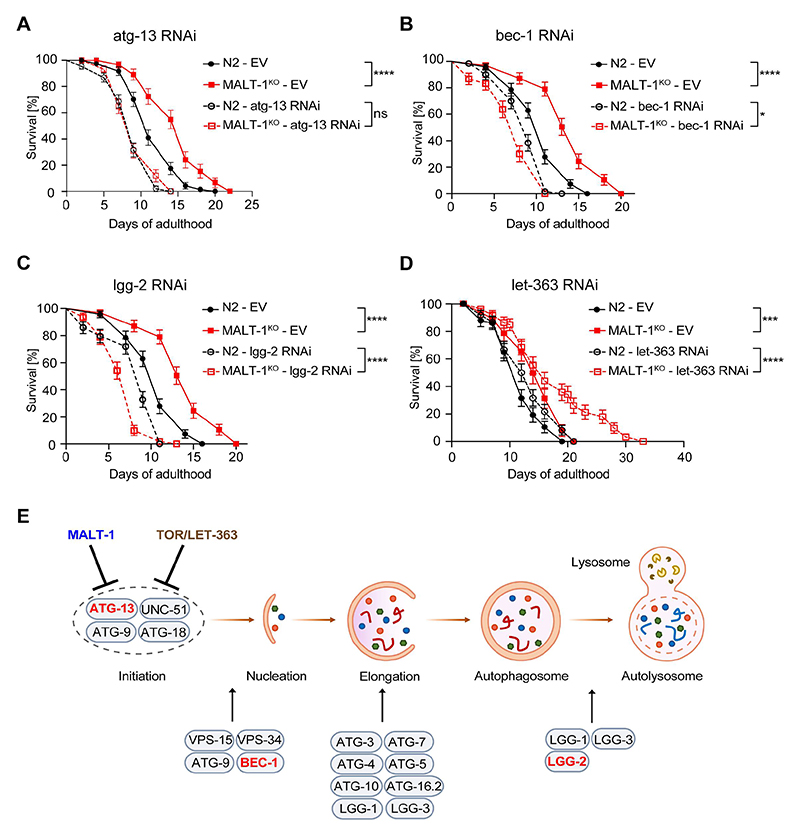
MALT-1 shortens longevity by inhibiting autophagy in a TOR independent manner. (A-D) Lifespan of N2 or MALT-1^KO^ strains grown from stage L4 on bacteria with control (empty vector, EV), atg-13 (A), bec-1 (B), lgg-2 (C) or let-363 (D) RNAi constructs. Graphs showing RNAi against bec-1 (B) and lgg-2 (C) were done in the same experiment and EV conditions are thus identical. The log-rank Mantel-Cox test was used for statistics. * p<0.05, *** p<0.001, **** p<0.0001, ns, not significant. Graphs are representative of two independent experiments for each condition. (E) Model for the inhibitory role of MALT-1 in the regulation of the initiation process of autophagy. *C. elegans* proteins that promote or inhibit individual steps are annotated. Genes targeted by RNAi in panels (A-C) are highlighted in red.

We reasoned that if the extended lifespan of MALT-1-deficient animals were indeed due to an overall increase in autophagic activity, it should be possible to overcome this effect by silencing the expression of key autophagy genes, such as ATG-13, BEC-1 and LGG-2 [36–38]. ATG-13, encoded by the *C. elegans* gene *epg-1*, is a homolog of mammalian and yeast Atg13 [38], which has an essential role in the induction of autophagy by binding and activating the kinase Atg1/ULK1 (encoded by *unc-51* in *C. elegans*) [39]. BEC-1, the *C. elegans* ortholog of mammalian Beclin-1, is a conserved core component of the PI3KC3 complex promoting phagophore nucleation [40]. LGG-2, a *C. elegans* homolog of the Atg8/LC3 family, is essential for a later step of autophagosome maturation by controlling autophagosome acidification [41,42]. To assess whether ATG-13, BEC-1 and/or LGG-2 were required for the life-span extension of MALT-1-deficient animals, we silenced the *atg-13/epg-1, bec-1, and lgg-2* genes in MALT-1^KO^ or N2 strains using RNAi, which in general targets all tissues except the nervous system. RNAi-based gene silencing efficiently downregulated *atg-13, bec-1* and *lgg-2* mRNAs (Figure S6A). The MALT-1^KO^ strain showed no significant changes in the relative mRNA levels of these autophagy regulators compared to the N2 wild-type strain (Figure S6B). Silencing of either one of the three autophagy regulators shortened the lifespan of *C. elegans*, consistent with previous reports [36,38,43]. Importantly, their silencing abolished the lifespan extension caused by MALT-1-deficiency, yielding similar lifespans for N2 and MALT-1^KO^ animals (Figure 5, A-C). We also tested the effect of treatment with chloroquine, a compound that blocks autolysosome acidification and thereby prevents the degradation of autolysosomal content (Figure S6C). As expected, if the MALT-1^KO^ strain had an increased level of autophagy, the KO animals were more sensitive to chloroquine treatment than wild-type animals, resulting in a similar survival curve for chloroquine-treated N2 and MALT-1^KO^ animals (Figure S6C). These findings support the notion that MALT-1 inhibits an early step in the autophagy process, and that genetic or chemical inhibition of various steps of autophagy can revert the life-span extension in the mutant MALT-1^KO^ strain.

### MALT-1 inhibits autophagy initiation independently of TOR

The fact that *atg-13* silencing abolished the lifespan extension conferred by MALT-1-deficiency suggested that MALT-1 directly or indirectly inhibits ATG-13-dependent autophagy initiation. In mammalian cells, ATG-13 is phosphorylated and activated by the UNC-51-like kinase ULK-1, which is itself inhibited by mTORC1 [44]. Nutrient limitation results in mTORC1 inactivation, releasing ULK-1 from inhibition and allowing ATG-13 phos-phorylation, thereby stimulating autophagy [44]. TOR signaling functions, including TORC1-mediated inhibition of autophagy, are conserved in *C. elegans* and contribute to the regulation of lifespan [44]. To test whether MALT-1 inhibits ATG-13-dependent autophagy by activating TOR, we treated N2 and MALT-1^KO^ animals with RNAi specific for the *C. elegans* TOR ortholog *let-363* and assessed their longevity. It was previously described that *let-363* silencing increases longevity [45–47] and we indeed observed a slight enhancement of survival when *let-363* gene expression was silenced in the N2 strain. Interestingly, *let-363* silencing considerably prolonged the lifespan of MALT-1^KO^ animals (Figure 5D). This contrasts with silencing *malt-1* or *let-363* individually, which had only a small effect, and suggests that MALT-1 and LET-363/TOR act on different, individually non-essential members of the autophagy complex, or perhaps are not completely penetrative on the same target protein/complex (Figure 5E).

We also explored a possible functional link between MALT-1 and the transcription factor DAF-16/FOXO. DAF-16 is strongly linked to longevity and promotion of autophagy by integrating signals from the insulin/IGF-1 signaling pathways and inhibiting expression of the essential TORC1 component DAF-15/Raptor [48,49]. To investigate whether MALT-1 and DAF-16 act in the same pathway, we crossed the *daf-16(mu86)* mutant strain with the MALT-1^KO^ or MALT-1^C372A^ strains and assessed their longevity (Figure S6D). We observed that MALT-1 deficiency or inactivation still further prolonged the lifespan of the *daf-16(mu86)* mutant, suggesting that DAF-16/FOXO and MALT-1 act independently.

Finally, we assessed whether the absence of MALT-1 led to an increase in the activation, e.g. nuclear translocation of transcription factors controlled by TORC1, such as HLH-30/TFEB [26,50]. HLH-30 translocated to the nucleus during a heat shock at 37°C and silencing of *malt-1* did not alter its subcellular distribution at 20°C or 37°C, when HLH-30 was exclusively cytoplasmic or nuclear (Figure S6E), nor at 28°C, a temperature at which HLH-30 partially translocated to the nucleus (Figure S6F). Moreover, rapamycin-induced translocation of HLH-30 to the nucleus was not affected by *malt-1* silencing (Figure S6F), suggesting that MALT-1 does not affect TORC1- and HLH-30 dependent cellular responses. In conclusion, these findings suggest that MALT-1 inhibits an early step in the autophagy process, upstream or at the level of ATG-13, independent of TORC1.

## Discussion

Here, we provide several lines of evidence for a role of MALT-1 in limiting the lifespan of *C. elegans* through inhibition of an early step of the autophagy process. The proposed role of MALT-1 in limiting longevity is specific to the intestine, since RNAi-mediated silencing or overexpression of MALT-1 in the intestine mimicked the lifespan changes observed in *C. elegans* that systemically lacked or overexpressed MALT-1. Consistent with an important role in the intestine, we detected expression of MALT-1 in the entire digestive tract, comprising the pharynx, the intestine, and the anal region, in addition to its previously reported expression in neurons [14].

The reasons for the opposing effects on lifespan of malt-1 RNAi in neurons (which yields a shorter lifespan [13,14] *vs* the intestine (where we saw a prolonged lifespan) are currently unclear. We cannot exclude that MALT-1 inhibits autophagy similarly in both organs. Such a hypothesis requires that increased autophagy benefits the intestine (through increased resistance to starving), yet impedes neuronal functions. The latter seems unlikely in view of prior studies that link increased autophagy in neurons to increased longevity [51,52]. We therefore favor an alternative hypothesis, namely that the effects of malt-1 RNAi on autophagy are specific to the intestine (for example through tissue-specific presence of adaptor/signaling proteins necessary for MALT-1-mediated inhibition of autophagy), whereas lack of MALT-1 in neurons shortens lifespan in an autophagy independent manner, through effects on neuronal IL-17 signaling [14].

Our data suggest that the catalytic activity of MALT-1 contributed to the extended lifespan of MALT-1-animals since both, MALT-1-deficient nematodes and animals with a protease-inactivating point mutation of MALT-1 had an extended lifespan. Moreover, animals overexpressing a protease inactive MALT-1 in a wild-type background showed an increase in longevity, whereas overexpression of active MALT-1 shortened the lifespan of the animals. These findings point towards the existence of *C. elegans*-specific MALT-1 substrates and binding partners that play a role in *C. elegans* aging. Interestingly, under starving conditions, the scaffold function of MALT-1 was more prominent than its protease activity in terms of lifespan regulation, suggesting that MALT-1 binding partners may play a prevalent role during starvation.

Autophagic processes, particularly in the intestine, are beneficial for increasing the lifespan of *C. elegans* during food deprivation [53–56]. Since MALT-1-deficient nematodes were much more resistant to food deprivation than the N2 strain, we explored the possibility of a role for MALT-1 in autophagy. Using two autophagy reporter strains and by TEM, we observed an increase of autophagosomes and autolysosomes in the intestine of MALT-1^KO^ animals compared to controls after starvation or during aging. These data suggest that MALT-1 inhibits the autophagic process. Consistently, we found that the increased longevity observed in MALT-1-deficient animals was abolished when different autophagic processes, including autophagy initiation, were genetically or chemically inhibited. These results support a model in which MALT-1 inhibits an early step of the autophagy process, independently of the TORC1/mTOR pathway, in *C. elegans* (Figure 5E).

It remains currently unclear whether MALT1 also inhibits autophagy in mammals. Two studies have reported a role for autophagy in downregulating CBM signaling in T cells and macrophages [57,58]. T cell activation by TCR stimulation leads to autophagic degradation of the MALT1 binding partner BCL10, but not of MALT1 itself [57]. In this context, MALT1 colocalizes with LC3 and autolysosomes, suggesting a physical link to the autophagic process. In macrophages, the autophagy inhibitor Rubicon directly interacts with the CBM complex component CARD9, promoting CBM dissociation and terminating signaling from pattern recognition receptors [58]. It is tempting to speculate that, in these contexts, MALT1 might inhibit autophagy to strengthen CBM signaling and boost immune responses. Finally, a highly interesting recent study exploring the role of MALT1 in glioblastoma has reported that MALT1 silencing or loss of MALT1 activity leads to an increase in the formation of cytoplasmic vacuoles and endo-lysosomes, as well as an accumulation of autophagic structures [59], which is similar to our observations. The accumulation of autophagic structures in the latter study was proposed to be the consequence of impaired autophagic flux rather than an increase in autophagic activity [59]. However, in the same study, other markers of autophagy were elevated, and how specific the autophagy changes are to neurons remains to be investigated. Altogether, it thus seems plausible that the increase in autophagic structures in MALT-1-deficient *C. elegans* intestinal cells results from an overall increase in lysosomal/vacuolar and autophagic activities that are normally restricted by MALT-1.

How exactly MALT-1 controls autophagy in *C. elegans* remains unclear. A previous study had suggested that in neurons, MALT-1 responds to IL-17 signaling by activating the transcription factor NFKI-1, a homolog of mammalian IκBζ and IκBNS [14]. This study also revealed that an over-lapping set of autophagy-regulating genes were upregulated in MALT-1-or NFKI-1-deficient animals. Although MALT-1-deficient animals showed no alteration in the expression of atg-13, bec-1, lgg-2 or let-363 mRNA in our hands, it remains possible that MALT-1 may, at least in part, regulate autophagy by affecting expression of other autophagy-regulating genes. In addition, MALT-1 may control autophagic processes by physically binding or cleaving autophagy regulators. The identification of the relevant MALT-1 binding partners and substrates, predicted to be specific to the intestine of *C. elegans*, will be the challenging topic of future studies.

Amongst known MALT-1 substrates in human and mice, only few have homologs in the nematode. These include two RNA regulators, REGE-1 (a ribonuclease related to mammalian Regnase-1 [60]), and RLE-1 (a homolog of mammalian Roquin-1/2 [61]), in addition to the USP domain-containing protein CYLD-1 (a homolog of the human deubiquitinase CYLD [62]). RLE-1, as its full name (Regulation of Longevity by E3 ubiquitin ligase) suggests, was described to shorten longevity by adding polyubiquitin on DAF-16, leading to its degradation by the proteasome [63]. In the scenario where MALT-1 would cleave RLE-1, this should result in a lifespan extension, and we would thus expect a decrease in longevity in the MALT-1^KO^ animals. Our results go however in the opposite direction, making it unlikely that RLE-1 is a MALT-1 substrate involved in aging. The two other candidates, REGE-1 and CYLD, have no known roles in longevity or autophagy in the nematode. Interestingly, in humans, CYLD and Regnase-1 have been previously proposed to play a role in autophagy in neurons and adipocytes, respectively [64,65], suggesting that they may be autophagy-relevant substrates in *C. elegans*. We attempted to test this possibility by co-expressing tagged forms of these proteins with active human MALT1 in 293T cells but did not detect signs of protein cleavage under these conditions (data not depicted). However, since *C. elegans* needs a living temperature below 30°C, we cannot exclude misfolding of the potential nematode substrate proteins at 37°C, the temperature used for mammalian cell culture. We also attempted to monitor substrate cleavage by *C. elegans* MALT-1 *in vitro* but were unable to generate active recombinant *C. elegans* MALT-1 protein, most likely because of lack of knowledge about required co-factors or posttranslational modifications. Indeed, human MALT1 requires monoubiquitination on K644 and the presence of an intact, negatively charged ubiquitin binding site comprising E696 and D697 within the Ig3 domain for its inducible activation [66,67]. Neither of these sites are conserved in *C. elegans* MALT-1, suggesting that the nematode uses a hitherto unknown mode of MALT-1 activation.

In conclusion, our findings reveal an unexpected role for MALT-1 in limiting intestinal autophagy, which has important consequences on the lifespan of *C. elegans*. Further studies investigating MALT-1 activators, such as the identification of cofactors, receptors and/or environmental events triggering MALT-1 activity, will be instrumental to gain additional insight into the role of MALT-1 in inhibiting autophagy. Nonetheless, this work has laid a solid foundation for such pursuits as it clearly establishes the position of MALT-1 at the initiation of the autophagic pathways and shows that loss of MALT-1 has physiologically relevant impacts on lifespan in one of the most venerable models of aging.

## Materials and Methods

### *C. elegans* strains and maintenance

Standard methods of culturing and handling *C. elegans* were followed [68]. *C. elegans* were maintained on standard nematode growth medium (NGM) plates streaked with OP50 *E. coli*. Strains used in this study were obtained from the *C. elegans* Genetics Center (University of Minnesota, MN, USA) or built as requested by Knudra (Salt Lake City, US) and Sunybiotech Co., Ltd (Fuzhou, China). Mutant strains were outcrossed at least two times with N2 before use, except for PHX6548 and PHX6791. Animals were picked onto new NGM plates without FUdR, every 2 days. Strains used in this study include: N2 Bristol strain, TU3401 *sid-1(pk3321)*;uIs69, MG171 *sid-1(qt9)*;aIxIs9, MTM6 (knu648 [C374A]) (referred to as MALT-1^C374A^), MTM12 *malt-1(syb492)* (referred to as MALT-1^KO^), MTM20 sySi61[dpy-30p::MALT-1::MALT-1 3’UTR]; *unc-119(ed3)* made by Sunybiotech, which overexpresses MALT-1 under the ubiquitious *dpy-30* promoter (referred to as MALT-1^OE^), MTM21 sybSi70[dpy-30p::MALT-1-C374A::MALT-1 3’UTR];*unc-119(ed3)* made by Sunybiotech, which overexpresses MALT-1^C374A^ under the *dpy-30* promoter (referred to as MALT-1^OE-C374A^) PHX6548 syb6548[rgef-1p::MALT-1::MALT-1 3’UTR] for MALT-1 overexpression in the nervous system (referred to as MALT-1^OE NS^), PHX6791 syb6791[ftn-1p::MALT-1::MALT-1 3’UTR] for MALT-1 overexpression in the intestine and, to a lower extent, in the pharynx (referred to as MALT-1^OE Int^), DA2123 adIs2122[lgg-1p::GFP::lgg-1 + rol-6(su1006)], MAH215 sqIs11[lgg-1p::mCherry::GFP::lgg-1 + rol-6], MTM38 sqIs11[lgg-1p::mCherry::GFP::lgg-1 + rol-6];*malt-1(syb492)*; CF1038 *daf-16(mu86)*; MTM14 *malt-1*(*knu648)* [C374A]; *daf-16(mu86)*, MTM15 *malt-1(syb492);daf-16(mu86)*, MAH240 sqIs17(hlh-30p:: hlh-30::GFP + rol-6(su1006). PHX629 *malt-1(syb629)*, which is GFP::MALT-1, was made by Sunybiotech. To facilitate microscopic detection of GFP::MALT-1 construct expressed under the control of its endogenous reporter, we crossed PHX629 with the JJ1271 *glo-1(zu391)* strain, MTM13 *malt-1(syb629);glo-1-(zu391)*. This strain is deleted for the *glo-1* gene encoding an ortholog of human RAB32 [27], and therefore lacks autofluorescent gut granules important for autophagy [69–71].

### RNAi experiments

RNA interference (RNAi)-treated strains were fed *E. coli* (HT115) containing an empty vector or ZK593.6 (lgg-2), D2007.5 (atg-13), T19E7.3 (bec-1) or B0261.2 (let-363). RNAi clones were bought from the worm ORFeome RNAi library (Horizon discovery, Cambridge, UK) and from K.K. DNAFORM. RNAi for malt-1 was constructed with the primers 3’-AAAGGGCCCGTTTACGTATTCGCATCAATA-5’, incorporating the Apa1 restriction site, and 3’-AAACTCGAGCTGTAGACATTTGATTCTTG-5’, with an Xho1 restriction site. The sequence targeting exon 14 of *malt-1* was cloned into the L4440 plasmid and transformed into HT115 competent bacteria. RNAi experiments were performed at 20°C. Nematodes were grown on NGM enriched with 1 mM isopropyl-D-thiogalactopyranoside (IPTG) and 50 μg/ml ampicillin. To minimize developmental effects, L4 animals were grown on plates with RNAi bacteria and assayed for paralysis as adults. Animals were transferred to fresh plates every 2 days.

### Lifespan

Between 60 and 90 animals were divided in triplicates on OP50-streaked NGM plates at L4 stage. Every two days all animals were counted and picked onto new plates to maintain synchronization. Nematodes that did not respond to the head touch assay were considered dead. For heat-killed bacteria, a pellet of OP50 was heated at 70°C for 1 h after two cycles of freeze-thaw in liquid nitrogen. NGM plates with low peptone concentration (2.5 mg/l instead of 2.5 g/l) were streaked with live OP50. For starvation assay, at day 2 of adulthood, synchronized animals were picked onto plates supplemented with ampicillin 0.25 mg/ml and without any bacteria. For inhibitor treatments, plates were supplemented with a final concentration of 20 mM chloroquine diphosphate salt (Sigma, C6628), or 100 μM rapamycin (Lucerna-Chem, #R-5000). All the experiments were performed without floxuridine (FUdR).

### PCR and RT-PCR assays

For PCR, Green Mix (Promega, M7122) was used following the manufacturer’s protocol. RNA samples were obtained from 15 confluent plates of animals, following Trizol reagent (Life Technologies, amphion, 15596026)/chloroform extraction and quantified with a Nanodrop 2000 spectrophotometer (Thermo Scientific). Five μg of RNA were used. Primers used include malt-1 (F22D3.6) forward 5’-CCCCACTAGCACTACTCCTCC-3’; malt-1 (F22D3.6) reverse GTACTTCGAATCTCGGGAACTTGAAG-3’; and malt-1 (F22D3.6) forward 5’-TATGGATACTCTACAAGCGGTGG-3’; malt-1 (F22D3.6) reverse 5’-GACCCGTAG GTGTTCACATGG-3’; *act-3* (T04C12.4) forward 5’-ATCCAAGCTGTCCTCTCCCTCTACG-3’; *act-3* (T04C12.4) reverse 5’-AGTTCCAGCTATGGTATATGCTC-3^’^; *lgg-2* (ZK593.6) forward, 5’-TCGTTCCATCGTTCAAGGAAAGAAGGCCATT-3’; *lgg-2* (ZK593.6) reverse, 5’-TTATATGCTCGGGTACTAGGAATTTGCAGCGA-3’; *bec-1* (T19E7.3) forward, 5’-TCACGAACTGATGCCCGTCGCCATGGGT-3’; *bec-1* (T19E7.3) reverse 5’-AGCCATTGCACGAGTCCATCGAACATCTGT-3’; *let-363* (B0261.2) forward 5’-TATAAGAAAGCACGCGGCACCGTATATGCCA-3’; *let-363* (B0261.2) reverse 5’-TTTGTAGTACAGTCAACAGATACGGTATTAGTTCGCCT-3’; *atg-13* (D2007.5) forward 5’- ACATTGCTTCGATCAGCAATTGTATCAGCCAGAA-3’; *atg-13* (D2007.5) reverse 5’- AGTCACTCATCGGAGAACGAATTGACGTGTT-3’.

### Progeny assay

Ten animals for each strain were picked on individual fresh OP50 plates at the L4 stage. Every day for 5 days eggs laid on plates were counted, and each adult was picked onto fresh plate. After 5 days, the total number of progenies per animal was calculated.

### Pharyngeal pumping record

Five animals for each strain were recorded with a Canon Camera mounted on Nikon binocular for 30s. The recording was processed with iMovie (version 10.1.6 Apple) to half the speed and allow counting the number of peristalses.

### High-pressure freezing and freeze substitution

Samples from the starved plates were collected with an aliquot of OP50 and placed in 0.2 µm thick aluminum carriers filled with 20% BSA. The carriers were covered with a lid and frozen under high pressure (~ 2000 bars) in a Wohlwend HPF Compact 02 (Wohlwend GmbH, Switzerland). The overall processing time did not exceed 5 min in all cases.

Freeze substitution was done in an AFS2 (Leica Mikrosysteme GmbH, Vienna, Austria) following the rapid substitution protocol modified from McDonald and Webb [72]. In brief, carriers were transferred to the freeze-substitution mix inside the liquid N_2_ storage and placed immediately in the AFS at -140°C. The mix of 0.1% (w/v) uranyl acetate in acetone was pre-cooled beforehand to -140°C. Afterwards, the temperature was elevated to -90°C over 6 h and further raised to 0°C over the next 18 h. Samples were left at this temperature for 1 h and then washed three times with acetone. After this step, samples were transferred to room temperature and infiltrated with increasing concentrations (30%, 50%, 75%, 4 h each) of EPON (EMS) in acetone. Finally, 100% EPON was exchanged three times in 10 h steps, samples were flat embedded and polymerized for 48 h at 60°C [73,74].

### Ultra-thin sectioning

Polymerized flat blocks were analyzed and carefully oriented under the microscope to obtain the desired orientation before the sectioning. Next, blocks were tightly trimmed using a 90° diamond trim tool and sectioned with 35° diamond knife (Diatome AG, Nidau, Switzerland), mounted on Leica UC6 ultramicrotome (Leica Mikrosysteme GmbH, Vienna, Austria). Finally, 70 nm sections were collected on polystyrene-coated slot grids (Electron Microscopy Sciences, Hatfield, PA, USA) [75].

### TEM imaging and analysis

The grids were analyzed with a Philips CM100 transmission electron microscope (Thermo Fisher Scientific, Waltham, MA, USA) at an acceleration voltage of 80 kV with a TVIPS TemCam-F416 digital camera (TVIPS GmbH, Gauting, Germany), piloted by the EMTVIPS program. A composite image containing 100 high-resolution images was collected for each sample section to cover the entire cross-section. These mosaic images were stitched using Blendmont command-line program from IMOD software [76].

### Stereology

The data were analyzed using 3D Mod software (Version 4.11.2) [76]. Pictures were divided into a grid spaced between 200 and 500 pixels in order to have more than 1000 intersections per image. Individual intersections of the grid were analyzed and assigned as mitochondria, cytosol, double membrane vesicles (autophagosomes), and other organelles such as Golgi, simple membrane vesicles, lipid vesicles, endoplasmic reticulum and uncertain structures. Statistical analyses were performed using a Mann-Whitney test.

### Confocal microscopy

*C. elegans* were synchronized and deposited at the indicated days into a drop of 60% glycerol on a 2% agarose pad. Pictures were taken on a confocal LSM800 Zeiss microscope. Intensity was fixed depending on the maximum fluorescence obtained. For time course experiments at days 3, 5 and 7 on MAH215 and MTM38, a reverse time course was performed to acquire all pictures at the same time. AP and AL quantifications were done in the anterior part of the intestine by ImageJ. In some cases, animals were starved for 96 h before imaging, as indicated. For the DA2123 images, malt-1 RNAi was applied for 24 h, then the animals were placed on a plate supplemented with 50 μg/ml ampicillin and without bacteria for 5 or 7 days according to the experiment. For HLH-30::GFP translocation analyses, we used L4 MAH240 animals, treated with EV or malt-1 RNAi at the indicated temperature, with or without 100 μM of rapamycin for 2 h. Then we immerged the nematodes on an agarose pad with a drop of 60% glycerol for confocal imaging. Diffuse fluorescence in an animal was considered as cytoplasmic, while punctate staining was scored as nuclear.

### Western blot

Protein samples were obtained from 15 confluent plates of non-synchronized animals, washed using M9 buffer and lysed in RIPA buffer, followed by three sonication cycles of 30 seconds on ice. Protein levels were quantified using Bradford reagent (Coomassie Plus, Thermo Scientific, #23238) and 20 µg was loaded on a 10% polyacrylamide gel, together with molecular weight standards (PageRuler, ThermoScientific, #11852124). Antibodies used include rabbit anti-GFP (1:1000, Sigma Aldrich, #G1544), mouse anti-Tubulin (1:1000, Cell Signaling, #3873) and secondary HRP-linked antibodies directed against mouse IgG (1:10,000, Cell Signaling, #7076S) and rabbit lgG (1:10,000, Cell Signaling, #7074S). HRP activity was revealed using ECL (Supersignal, West Atto Ultimate, ThermoScientific, #A38554). Anti-GFP was used to monitor expression of LGG-1 in the MAH215 and MTM38 strains.

## Supplementary Material

Suppl Material

## Abbreviations

AP-1: activator protein-1
AL: autolysosome
AP: autophagosome
ATG: Autophagy-related gene
BCL10: B cell lymphoma-10
CARD: caspase-recruitment domain
CARMA: CARD-MAGUK
*C. elegans*: *Caenorhabditis elegans*
CBM complex: CARMA-BCL10-MALT1 complex
EGFR: epidermal growth factor receptor
ER: endoplasmic reticulum
EV: empty vector
GFP: green fluorescent protein
GPCRs: G-protein coupled receptors
Ig: Immunoglobulin
Int: intestinal
ITAMs: immunoreceptor tyrosine-mediated activation motifs
KO: knock-out
LC3: microtubule-associated protein 1A/1B-light chain 3
LUBAC: linear ubiquitin chain assembly complex
MALT-1: mucosa-associated lymphoid tissue protein-1
NF-κB: nuclear factor-kappa B
NFKI: NF-κB inhibitor
NGM: nematode growth medium
ns: not significant
NS: nervous system
OE: overexpressed
RER: rough endoplasmic reticulum
RNAi: RNA interference
TCR: T cell receptor
TEM: Transmission electron microscopy
TRAF6: TNF receptor-associated factor-6.
