# Supplementary material for: MALT-1 shortens lifespan by inhibiting autophagy in the intestine of C. elegans
Source: Autophagy Rep. Author manuscript; Available in PMC 2024 Mar 20. (PMC7615756; doi:10.1080/27694127.2023.2277584)
Supplement: Suppl Material [file EMS192722-supplement-Suppl_Material.pdf]

Supplementary information

MALT-1 shortens lifespan by inhibiting autophagy in the intestine of *C. elegans*

Julie Vérièpe[a,*], Silvia Podavini[a], Marcus J.C. Long[a], Irina Kolotuev[b], Muriel Cuendet[c,d], Margot Thome[a].

[a] Department of Immunobiology, Faculty of Biology and Medicine, University of Lausanne, Chemin des Boveresses 155, CH-1066 Epalinges, Switzerland; [b] Electron Microscopy Facility, University of Lausanne, Quartier Sorge – Biophore, CH-1015 Lausanne, Switzerland, [c] School of Pharmaceutical Sciences, University of Geneva, Rue Michel-Servet 1, CH-1211 Geneva, Switzerland, [d] Institute of Pharmaceutical Sciences of Western Switzerland, University of Geneva, Rue Michel-Servet 1, CH-1211 Geneva, Switzerland

[*] Present address: School of Pharmaceutical Sciences, University of Geneva, Rue Michel-Servet 1, CH-1211 Geneva, Switzerland

Address correspondence to Margot Thome:

Dr. Margot Thome

Department of Immunobiology, University of Lausanne

Chemin des Boveresses 155, CH-1066 Epalinges, Switzerland

Phone: +41-21-692.57.37

Fax: +41-21-692.57.05

E-mail: Margot.ThomeMiazza@unil.ch

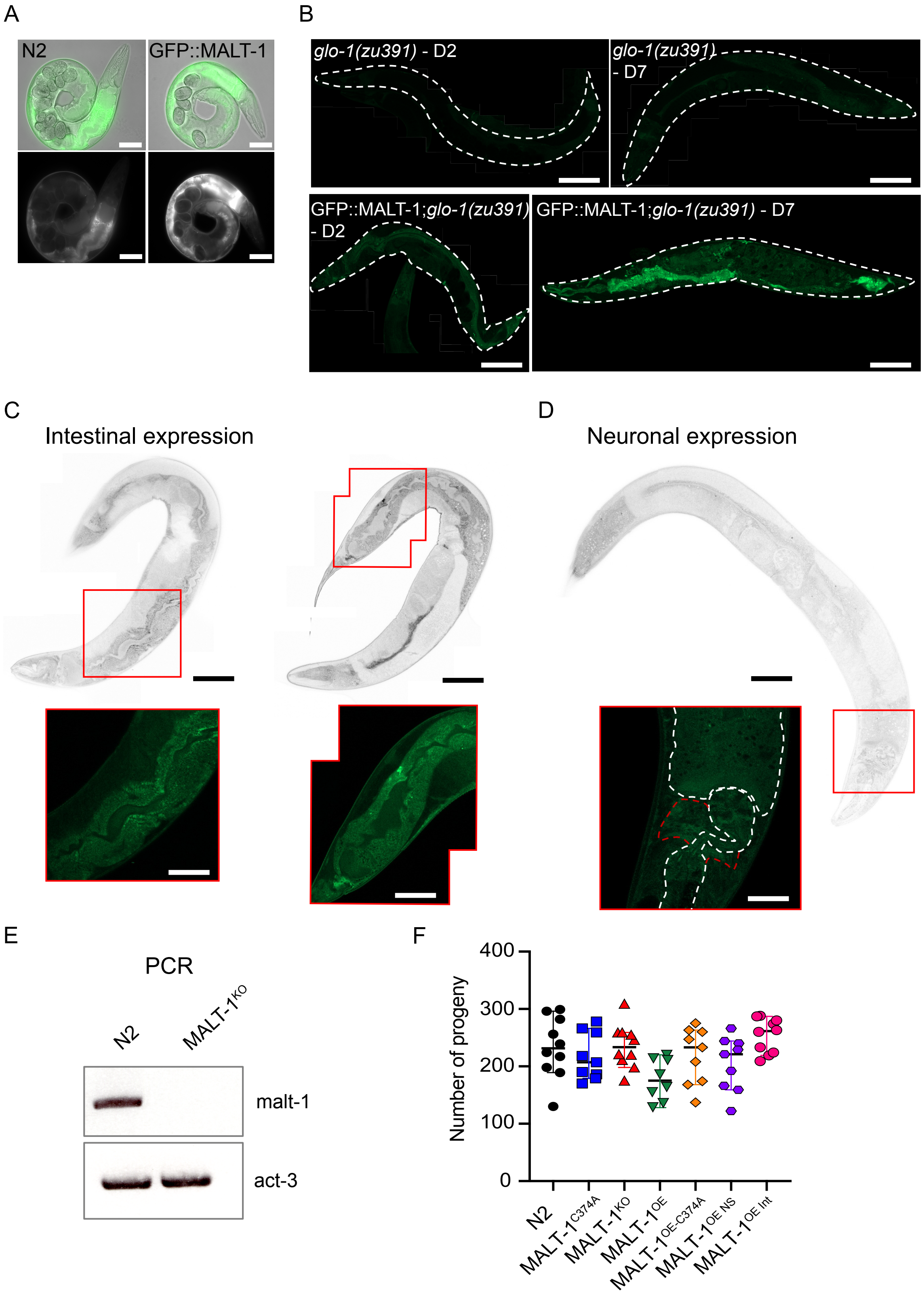

Figure S1. Characterization of strains expressing GFP::MALT-1 and of strains with altered MALT-1 expression or function.

(**A**) Fluorescence microscopy pictures of animals at day 3 of adulthood, comparing autofluorescence of N2 and fluorescence of the GFP::MALT-1 construct expressed under the endogenous MALT-1 promoter. Scale bar: 100 µm. (**B**) Confocal microscopy of GFP::MALT-1 crossed to *glo-1* mutant (GFP::MALT-1; *glo-1(zu391)*) at days 2 and 7 of adulthood. Note that glo-1(zu391), which is shown for comparison, has no or little autofluorescence. White dotted line indicates the contour of an animal. Scale bar: 100 µm. (**C-D**) Confocal pictures taken with the 63x oil objective. The whole animal was reconstructed and depicted in black and white for better contrast. A zoomed section of the intestine (**C**) or the nervous system (**D**) shows GFP::MALT-1 fluorescence in the selected region. Because the intestine crossed with the germline, the intestine is shown in two different Z-stack portions (anterior on the left, posterior on the right). For clarity, the shape of the pharynx is indicated by a white dotted line and of the neural nuclei by a red dotted line. Scale bars next to the whole animals: 80 µm, scale bars in the zoomed sections: 20 µm. (**E**) RT-PCR analysis of mRNA from N2 and MALT-1^{KO} to verify the absence of the mRNA in the knock-out strain. The housekeeping gene *act-3* was used as a control. (**F**) Analysis of the progeny number of the indicated strains. Each dot represents progeny number from a single nematode. Between 8 and 10 animals were analyzed. Statistical analysis was performed using unpaired t-test. Data are representative of two independent experiments.

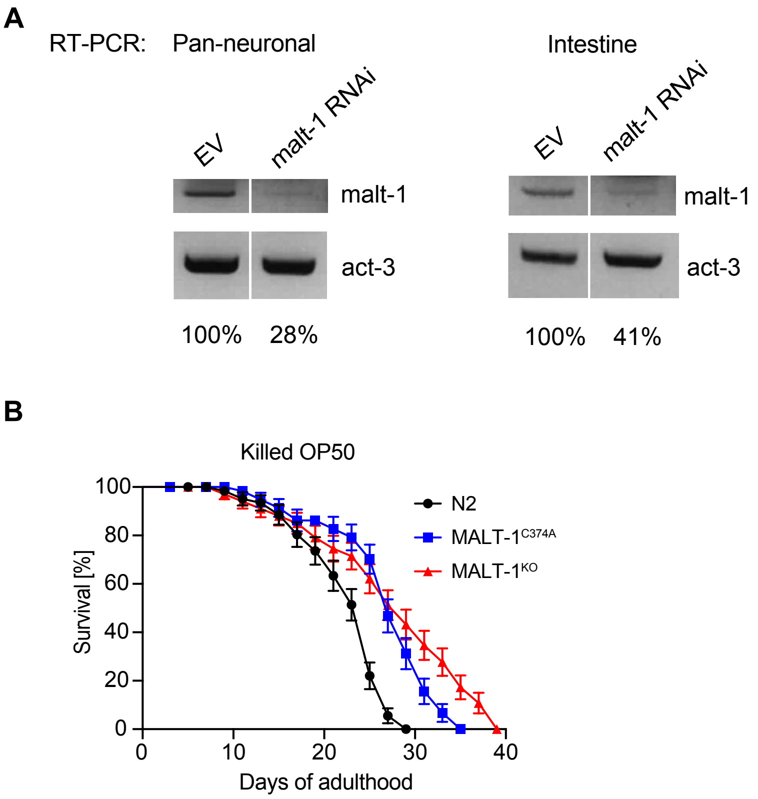

Figure S2. Characterization of the role of MALT-1 in the intestine and food pathogenicity. (**A**) RT-PCR analysis of malt-1 and act-3 mRNA levels to control the efficiency of malt-1 RNAi on strain TU3401 (pan-neuronal silencing, left panel) and strain MGH171 (intestinal silencing, right panel). Percentage of inhibition is indicated. (**B**) Lifespan of N2, MALT-1^{C374A} and MALT-1^{KO} animals on plates streaked with heat-killed OP50 to diminish bacterial pathogenicity.

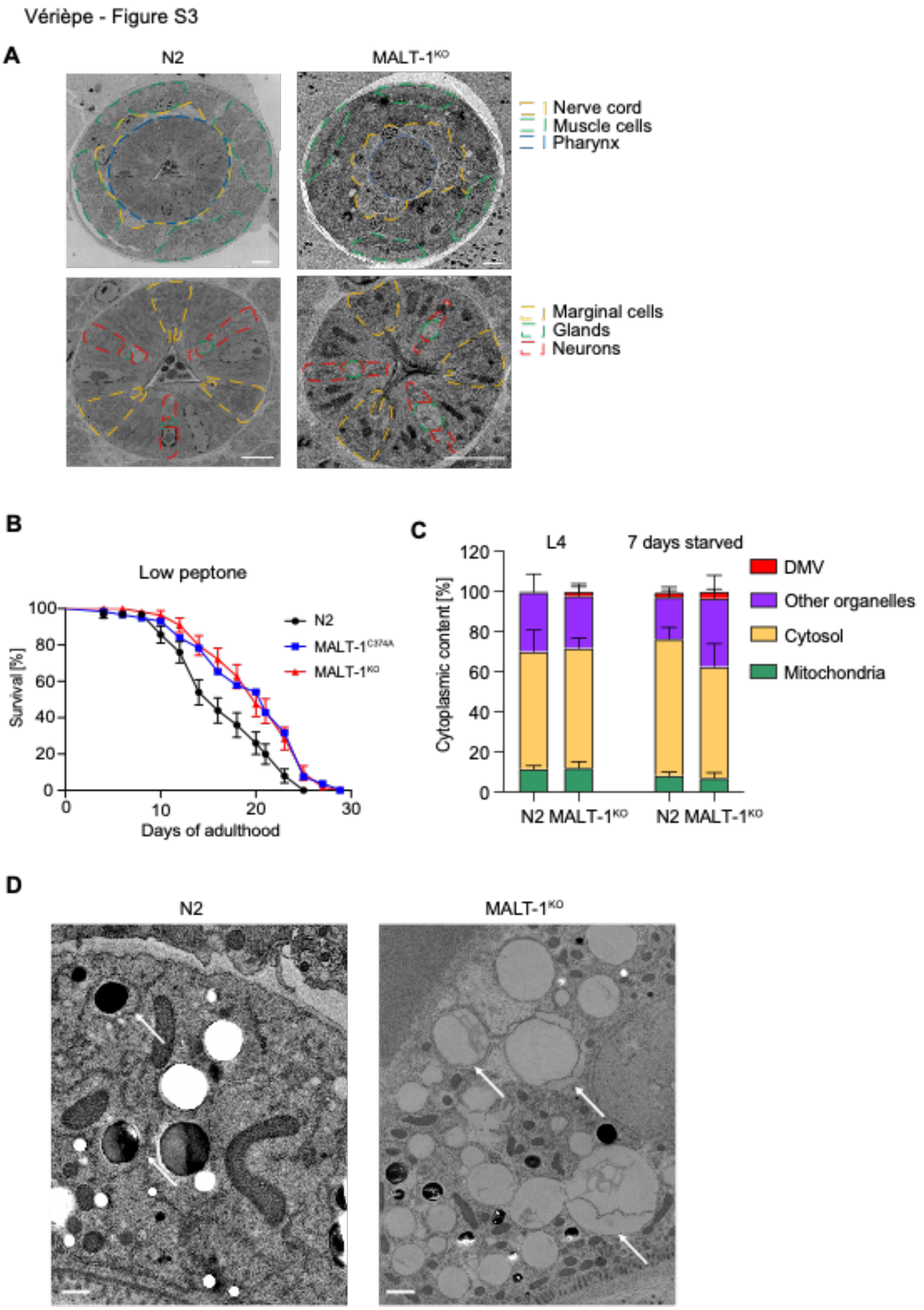

Figure S3. Electron microscopy analysis and response to starving of N2 and MALT-1^{KO} strains.

(**A**) Electron microscopy pictures of N2 (left) and MALT-1^{KO} (right), showing the pharynx (bottom panel) and the surrounding annotated structures (upper panel), revealing no obvious morphological alterations of the pharyngeal region of MALT-1^{KO} animals. Scale bar: 3 µm. (**B**) Lifespan of N2, MALT-1^{C374A} and MALT-1^{KO} strains on plates streaked with alive OP50 under low peptone conditions, to mimic restriction of food availability. Data are representative of three independent experiments performed. Log-rank Mantel-Cox statistics was used and p-values were <0.001 for each mutant compared to N2. (**C**) Stereological quantifications of pictures from three N2 and four MALT-1^{KO} animals at the L4 larva stage or after starving for 7 days (right part is identical to Figure 3E). Percentages of double membrane vesicles (DMV), mitochondria, cytosol and other organelles per image of intestinal cells are indicated (excluding intestinal lumen and nuclei). (**D**) Electronic pictures of N2 and MALT-1^{KO} strains revealing intriguing alterations of the structure of the endoplasmic reticulum in MALT-1^{KO} animals (ER structures are indicated with arrows). Scale bar: 1 µm.

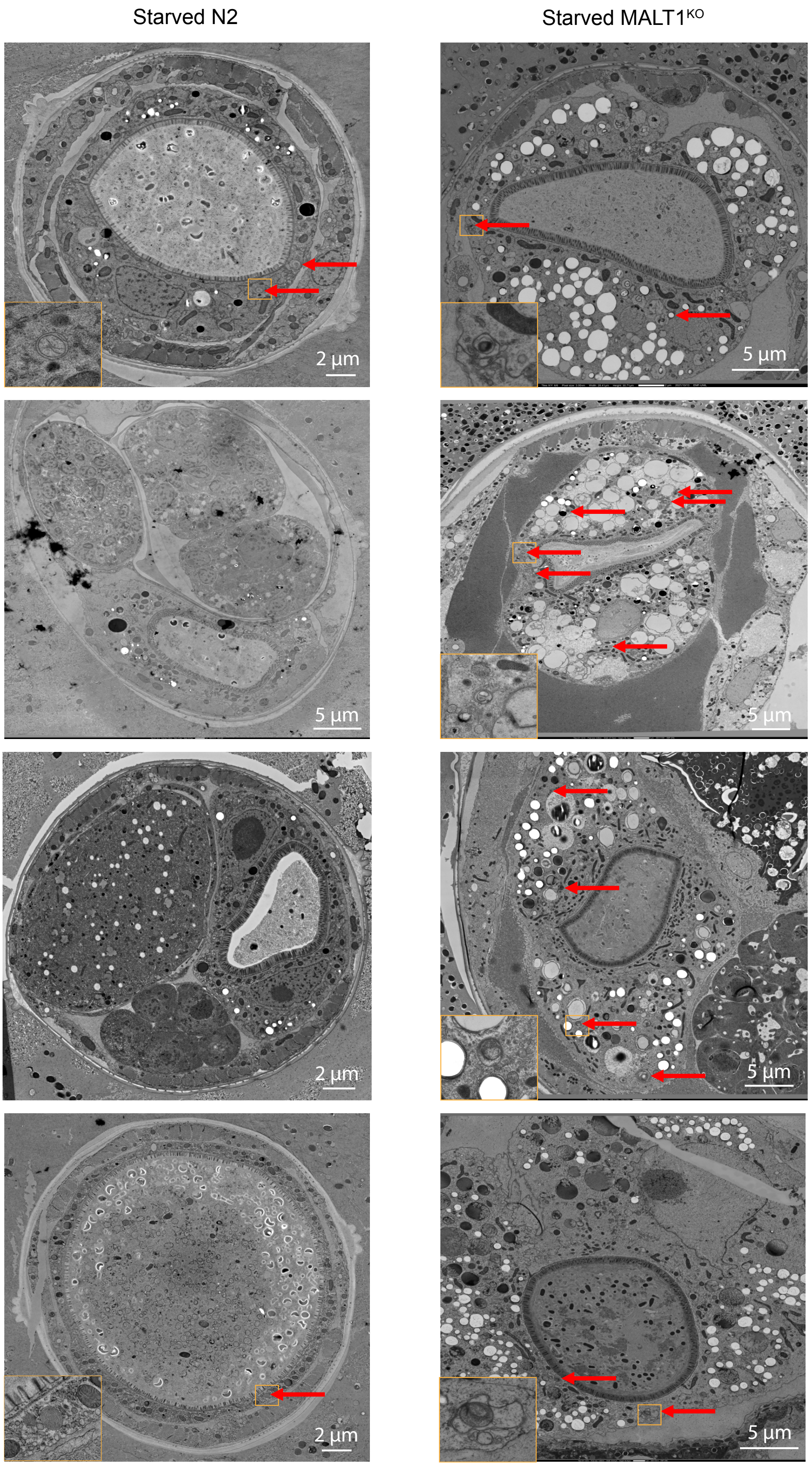

Figure S4. Electron microscopy images of the intestine of starved N2 and MALT-1^{KO} strains.

Red arrows indicate presence of double membrane vesicles in representative pictures of intestinal cells obtained from three N2 and four MALT-1^{KO} animals, used for quantification of double membrane vesicles in Figure 3F. Where appropriate, inset shows a magnification of one of the indicated vesicles.

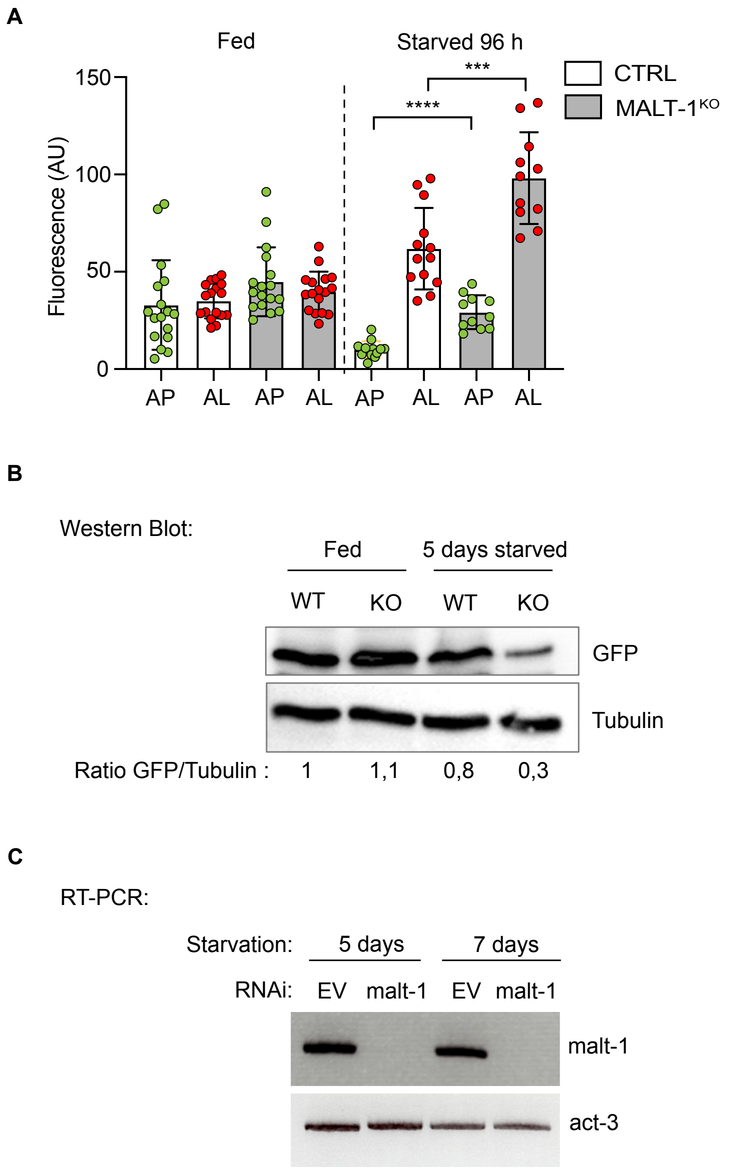

Figure S5. Increased autophagy in starved MALT-1-deficient animals.

(**A**) Quantification of control *vs* MALT-1^{KO} fluorescence intensities in nematodes starved for 96h (refers to Figure 4F). Relative fluorescence intensity of autophagosomes (AP) and autolysosomes (AL) is indicated by green and red dots, respectively, in fed and starved animals. AU, arbitrary units. Unpaired t-test was used for statistical analysis, ***, p=0.0006; ****, p<0.0001. (**B**) Analysis of the expression of an mCherry::GFP::LGG-1 fusion protein in the WT (MAH215) or MALT-1^{KO} (MTM38) background by western blot, using anti-GFP.

Asynchronized animals, fed or starved for 5 days, were collected for the MAH215 or MTM38 strains. Tubulin was used as a loading control and the relative ratios of the intensities of GFP/tubulin was quantified. (**C**) RT-PCR on mRNA extracted from GFP::LGG-1 animals, treated for 24 h with EV or *malt-1*-specific RNAi and then starved for 5 or 7 days, as indicated. Efficiency of *malt-1* silencing over the full starvation period was verified by monitoring malt-1 mRNA levels in comparison to act-3 mRNA, which represents expression of a housekeeping gene.

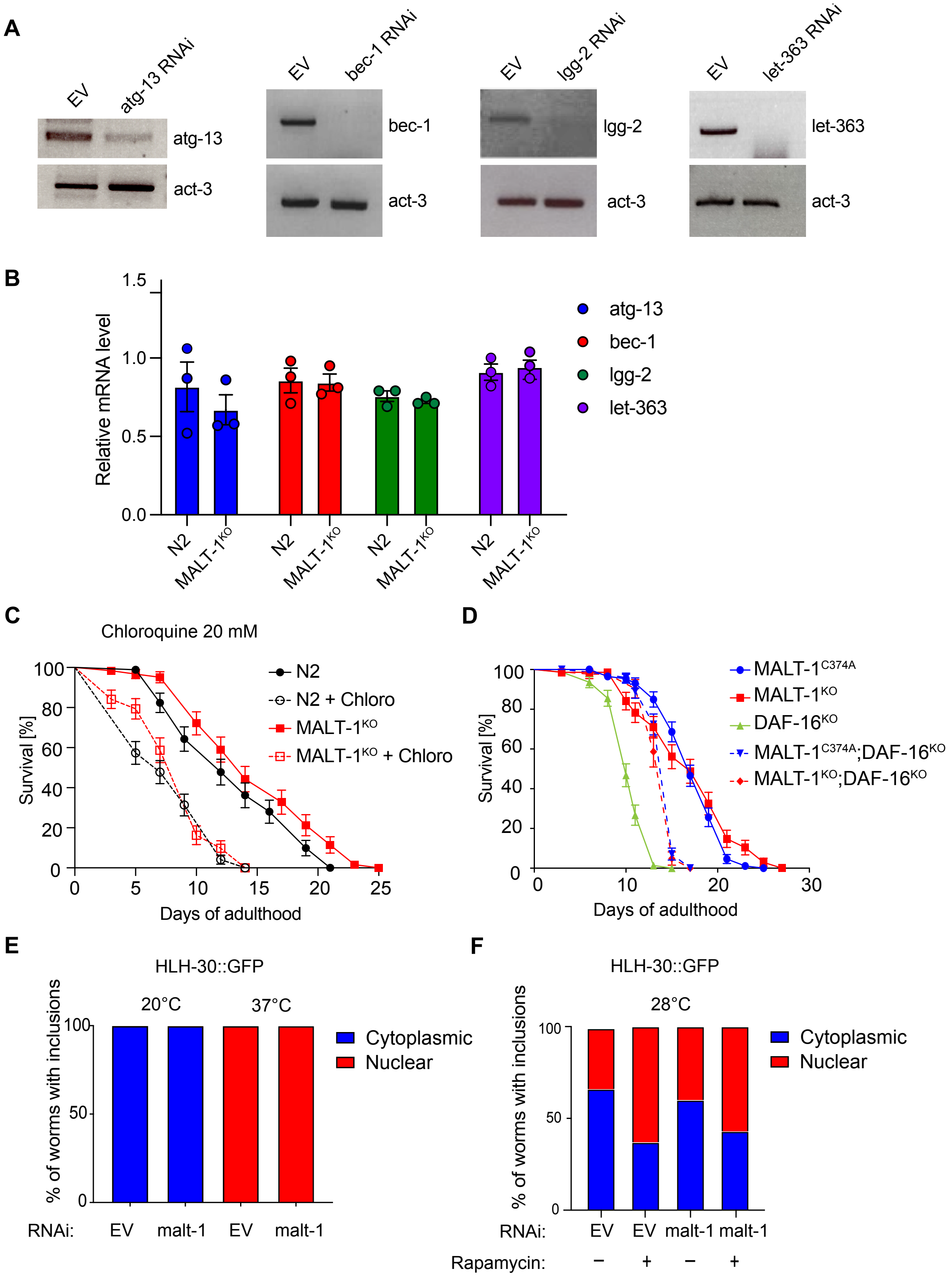

Figure S6. Effects of genetic and pharmacological manipulation of autophagy processes on the lifespan of N2 and MALT-1-deficient animals.

(**A**) RT-PCR analysis of agt-13, bec-1, lgg-2 and let-363 mRNA after RNAi-mediated gene silencing on the N2 strain. act-3 was used as a mRNA of a housekeeping gene. (**B**) Relative mRNA levels of autophagy-related genes, normalized to act-3 mRNA, in N2 vs MALT-1^{KO} strains. Experiments were done in triplicate, revealing no significant difference (unpaired t-test realized). (**C**) Lifespan of N2 and MALT-1^{KO} strains grown in the presence or absence of 20 mM chloroquine (chloro). The log-rank Mantel-Cox test was used for statistics. P-values were <0.05 only for the curves N2 vs MALT-1^{KO} without chloroquine treatment. (**D**) Lifespan of MALT-1^{C374A}, MALT-1^{KO}, DAF-16^{KO} and their crosses on OP50 at 20°C. Note that MALT-1 deficiency or catalytic inactivation of MALT-1 prolonged the lifespan of DAF-16^{KO}. (**E**) Percentage of HLH-30::GFP nematodes with cytoplasmic or nuclear inclusions of HLH-30::GFP at 20°C or after exposure to 37°C for 2 h. Gene silencing was performed with empty vector (EV) or *malt-1*-specific RNAi. (**F**) Percentage of HLH-30::GFP animals with cytoplasmic or nuclear inclusions after treatment with rapamycin (100 µM) at 28°C, upon gene silencing with empty vector (EV) or *malt-1*-specific RNAi, as indicated. Note that rapamycin treatment induced a similar increase in the proportion of nuclear HLH-30::GFP under EV or *malt-1* RNAi conditions. Data are representative of three (**D**) and two (**E, F**) independent experiments.

Supplementary tables

Strains	Median lifespan [days] (50% population dead)	Change in median lifespan [%]	Maximum lifespan [days]	Change in maximum lifespan [%]
Wild-type	11.5 ± 1.5		19 ± 0	
MALT-1$^{OE\ NS}$	15 ± 1	+ 31.5 %	25 ± 1	+ 31.5 %

Table S1 summarizing lifespan changes of a strain overexpressing MALT-1 in the nervous system (MALT-1$^{OE\ NS}$; PHX6791) compared to N2 (wild-type), calculated from two independent experiments.

Strains	Median lifespan [days] (50% population dead)	Change in median lifespan [%]	Maximum lifespan [days]	Change in maximum lifespan [%]
Wild-type	13 ± 1		20 ± 1	
MALT-1$^{OE\ Int}$	10 ± 1	- 19.6 %	20 ± 1	0 %

Table S2 summarizing lifespan changes of a strain overexpressing MALT-1 in the intestine (MALT-1$^{OE\ Int}$; PHX6548) compared to N2 (wild-type), calculated from two independent experiments.

Strains	Median lifespan [days] (50% population dead)	Change in median lifespan [%]	Maximum lifespan [days]	Change in maximum lifespan [%]
Wild-type	13 ± 2		21 ± 3	
MALT-1^{C374A}	17 ± 2	+ 30.7 %	26 ± 3	+ 23.8 %
MALT-1^{KO}	17 ± 2	+ 30.7 %	26 ± 3	+ 23.8 %
MALT-1^{OE}	9 ± 1	- 30.7 %	16 ± 2	- 23.8 %
MALT-1$^{OE-C374A}$	16 ± 1	+ 23.0 %	28 ± 0	+ 33.3 %

Table S3 summarizing lifespan changes in the indicated strains compared to N2 (wild-type), calculated from three independent experiments.
